# Vulvodynia Is Not Associated with Concurrent Candidal Vaginitis

**DOI:** 10.1089/whr.2021.0089

**Published:** 2022-02-02

**Authors:** Margaret Whitney, Amy E. Papermaster, Audrey Baum, Michelle L. Wright

**Affiliations:** ^1^ Department of Women's Health, The University of Texas at Austin Dell Medical School, Austin, Texas, USA.; ^2^ Advanced Practice Division, The University of Texas at Austin School of Nursing, Austin, Texas, USA.; ^3^ Adult Health Division in School of Nursing, The University of Texas at Austin School of Nursing, Austin, Texas, USA.

**Keywords:** vulvodynia, vulvar pain, vulvovaginal candidal infections, candida-positive vulvovaginal genital cultures

## Abstract

***Objective:*** The study purpose was to determine the prevalence of candida-positive vulvovaginal genital cultures among women with vulvodynia.

***Methods:*** This study was a retrospective analysis of data collected from 2017 to 2020. Eligible patients receiving care from an academic women's health practice in central Texas that employed value-based care pathways and who had a genital culture diagnostic test collected were included. Data were extracted from the electronic health record. Descriptive statistics, *t*-tests, and Fisher's exact test were used to complete the data analysis.

***Results:*** A total of 242 women met inclusion criteria and were included in the study. Of these, 64 (26.4%) had been diagnosed with vulvodynia and 178 (73.6%) had not. Of the 242 women, nearly one-third had confirmed yeast infections (29%) and 27 women (11%) met pathway criteria for polymerase chain reaction testing. There was no difference in the number of women with confirmed yeast infections during the study period among patients with or without a diagnosis of vulvodynia (75% vs. 70%, *p* = 0.718). Notably, among participants with vulvodynia, body mass index (BMI) was lower, and anxiety was more likely (*t* = 2.65, *df* = 120, *p* = 0.009; 78% vs. 55%, *p* = 0.002).

***Conclusions:*** The findings in this study showed no association between vulvodynia and yeast infection, a divergence from prior studies. In addition, vulvodynia was associated with low BMI and anxiety. Further research is needed to better understand the association between vulvovaginal candida infections and vulvodynia. Including women within and across more diverse races and ethnicities would improve generalizability.

## Introduction

The prevalence of vulvodynia, or persistent vulvar pain without identifiable etiology, is not well known. However, studies based on self-reported vulvodynia have estimated prevalence of 2%–15% in the United States.^1,2^ The cost of care for these women represents a significant financial burden, with recent cost estimates close to $9600.00 per treatment course in the United States.^3^ Women who present with vulvodynia symptoms frequently suffer from other costly and care-intensive chronic pain syndromes, such as fibromyalgia and painful bladder syndrome/interstitial cystitis.

The sexual and psychological health of these women are also negatively impacted.^4^ Current treatments are centered on symptom management and vary significantly due to the lack of robust data outlining the underlying pathophysiology of vulvodynia, which limits development of novel treatment. Identifying a clear etiology could reduce costs, improve treatment algorithms, and identify potential targets for new prevention strategies.

The etiology of vulvodynia has been the focus of a wide range of recent studies exploring potential environmental, psychological, and biological factors, from the use of hairspray to variations in the vaginal microbiome.^5,6^ More recently, interest in a possible relationship between vulvovaginal candida (VVC) infections and vulvodynia has emerged as an area of interest.

One current hypothesis for the etiology of vulvodynia includes candida infections that may initiate a local inflammatory response and subsequent vulvar pain.^7^ A systematic review of the literature looking at the relationship between localized provoked vulvodynia (LPV) and VVC was unable to determine any firm conclusions due to the fact that the studies relied on self-reported VVC.^2^ However, Falsetta et al.^8^ found that >70% of women with LPV with biopsies were positive for *Candida albicans* inflammatory markers and also reported having chronic VVC infection.

Taken together, these studies suggest a link between VVC infection and vulvodynia, but neither included laboratory confirmation of candida. Furthermore, 8% of all women suffer from recurrent VVC infection.^9^ The purpose of our study was to determine the prevalence of candida-positive vulvovaginal genital cultures among women who presented with vulvar pain and were given a subsequent vulvodynia diagnosis, compared with healthy controls.

## Methods

### Study design and participants

This was a retrospective analysis of the collected data collected from 2017 to 2020. Data were derived from an electronic health record (EHR). Eligible patients were those receiving care from an academic women's health practice in central Texas that employed value-based care pathways for complex gynecological conditions, including vulvodynia. The study was approved by the applicable university institutional review board (No. 2020-01-0129). There were no known risks to participation in the study and no compensation for participation in the study.

The participants were patients who had been seen at the university clinic and had signed a consent to have personal health information used for purposes of research. All study data were deidentified and kept on an encrypted password computer server (university approved Box©). All investigators who conducted data extraction were trained in the protection of human subjects involved in research and Health Insurance Portability and Accountability Act compliance.

Data were originally collected during routine clinical care of patients presenting for both routine and specialty vulvar care as part of a standardized value-based care pathway. The pathway utilized the 2015 International Society for the Study of Vulvovaginal Disease (ISSVD) criteria including the q-tip test and persistent vulvar pain without identifiable etiology, to establish a diagnosis of vulvodynia. Agar-based genital yeast culture was a standard component of the initial work-up for all patients presenting with vulvar pain as part of the value-based care pathway.

Polymerase chain reaction (PCR) testing was used in a minority of patients who met criteria for PCR testing per the pathway. In patients whose pain persisted after treatment of yeast and who received laboratory confirmation of resolution, the diagnosis of vulvodynia was made, in accordance with ISSVD diagnostic criteria. If persistent yeast was identified, the patients were included as part of a different clinical pathway for recurrent yeast infections.

Validated questionnaires for patient-reported outcome (PRO) measures including the Vulvar Pain Functional Questionnaire, Female Sexual Function Index, Generalized Anxiety Disorder (GAD-7), and Patient Health Questionnaire 9 (PHQ-9) were administered as part of routine patient intake. Medical records from December 18, 2017, to January 17, 2020, were reviewed. Participants under the age of 18 years were excluded.

A report was generated for the women's health clinic for agar-based genital culture and PCR diagnostic tests were collected. Data were extracted from the EHR using a standardized medical abstraction form and entered into a Microsoft Excel spreadsheet. Missing data fields were manually extracted from the individual health records. If PCR cultures were collected, the yeast speciation was delineated as *C. albicans* or nonalbicans species.

Patients with a diagnosis of vulvodynia were identified using diagnostic codes using the *International Classification of Disease*, 10th revision (ICD-10). Age, race, ethnicity, language, smoking, parity, body mass index (BMI), and diabetes were auto-populated fields. Anxiety and depression were classified per patient report of diagnosis, ICD-10 diagnosis, and PRO measurement screening using validated scales^10^ and PHQ.^11^ Additional variables included patient-reported dysmenorrhea, back pain, joint pain, fibromyalgia, history of sexually transmitted infection, type of contraception, and whether sexually active.

### Data analysis

Descriptive statistics were completed to analyze baseline differences among women with versus without vulvodynia, *t*-tests were used for continuous variables, and Fisher's exact test was used to assess categorical variables. Parity was categorized into 0, 1, 2, or 3 or more; birth control was categorized as hormonal (oral contraceptive pills, Depo Provera, *etc*.), surgical (hysterectomy, tubal ligation, *etc*.), menopause, or other (partner vasectomy, none, *etc*.).

The main goal of this study was to determine whether laboratory-confirmed yeast infection, either by agar-based culture or PCR, was associated with vulvodynia diagnosis. We included factors significantly different among women with versus without vulvodynia into a logistic model to examine likelihood of diagnosis by patient characteristics. All analyses were conducted using R statistical environment version 3.6.1 and an alpha of 0.05 was used to determine significance for all analyses.

## Results

A total of 242 women met inclusion criteria and were included in the study. Of these, 64 (26.4%) were diagnosed with vulvodynia and 178 (73.6%) were not. There was no difference in the number of women with confirmed yeast infections with or without a diagnosis of vulvodynia (75% vs. 70%, *p* = 0.718). Nearly one-third of women in the study had confirmed yeast infections (29%) and 27 women (11%) met pathway criteria for PCR testing. Among the patients who met pathway criteria for PCR testing, *C. albicans* accounted for 20 (75%) infections and the other 7 (25%) were nonalbicans.

A total of 73% self-identified as white, and 30% identified their ethnicity as Hispanic. The median BMI of our patient population was 26.7 kg/m^2^ and most reported never smoking (77%). Nearly half of our participants were nulliparous (46%) and sexually active (65%). Our patents reported 29 different varieties of contraceptive use, which were categorized into hormonal (22%), surgical (17%), menopause (18%) or other (42%). A comparison of baseline characteristics between patients with and without vulvodynia is presented in Table 1.

**Table 1.** Characteristics of Women According to Vulvodynia Diagnosis

Characteristic	All participants (***N*** = 242), ***n*** (%)	No vulvodynia (***N*** = 178), ***n*** (%)	Vulvodynia (***N*** = 64), ***n*** (%)	***p***
Age				
Minimum	14.00	18.00	14.00	0.698
Median (IQR)	39.00 (30.00–50.00)	39.00 (31.00–50.00)	38.50 (27.75–53.00)	
Mean (SD)	41.4 ± 14.3	41.47 ± 14.1	40.64 ± 14.7	
Maximum	85	85.00	75.00	
BMI (kg/m^2^)				
Minimum	16	17.90	16.00	0.009
Median (IQR)	26.70 (22.50–30.90)	27.30 (23.30–31.60)	24.95 (22.05–28.12)	
Mean (SD)	27.6 ± 6.83	28.33 ± 6.88	25.80 ± 6.38	
Maximum	61.00	61.00	48.60	
Unknown		5 (3)		
Race				
White	178 (73)	129 (72)	49 (77)	0.800
Black or African	20 (8)	13 (7)	7 (11)	
American				
Hispanic	6 (3)	5 (3)	1 (2)	
Asian	9 (4)	8 (4)	1 (2)	
Patient declined/missing	29 (12)	23 (13)	6 (9)	
Ethnicity				
Hispanic/Latinx	73 (30)	61 (34)	12 (19)	0.053
Non-Hispanic/Latinx	149 (62)	102 (57)	47 (73)	
Patient declined	20 (8)	15 (8)	5 (8)	
Parity				
0	112 (46)	78 (44)	34 (53)	0.059
1	31 (13)	23 (13)	8 (13)	
2	49 (20)	33 (19)	16 (25)	
3 or more	49 (20)	43 (24)	6 (9)	
Missing	1	1		
Birth control				
Hormonal	54 (22)	39 (22)	15 (23)	0.933
Surgical	42 (17)	31 (17)	11 (17)	
Menopause	42 (18)	30 (17)	12 (19)	
Other	103 (42)	78 (44)	26 (41)	
Recent culture				
*Candida albicans*	20 (8)	13 (7)	7 (11)	0.422
Non-*C. albicans*	7 (3)	5 (3)	2 (3)	0.657
Smoking				
Never	186 (77)	134 (75)	52 (81)	0.2542
Former	38 (16)	32 (18)	6 (9)	
Some days	9 (4)	7 (4)	2 (3)	
Daily	9 (4)	5 (3)	4 (6)	
Diabetes (% yes reported)	21 (9)	16 (9)	5 (8)	1
Anxiety	148 (61)	98 (55)	50 (78)	0.002
Depression	93 (38)	62 (35)	31 (48)	0.072
Period pain	89 (37)	66 (37)	23 (34)	0.919
Back pain	18 (7)	14 (8)	4 (6)	0.586
Joint pain	19 (8)	10 (6)	9 (14)	0.066
Fibromyalgia	7 (3)	3 (2)	4 (6)	0.146
STIs	53 (22)	38 (21)	15 (23)	0.871
Sexually active	157 (65)	116 (65)	41 (64)	0.961

BMI, body mass index; IQR, interquartile range; SD, standard deviation; STIs, sexually transmitted infections.

BMI was significantly lower among participants with vulvodynia than among those without (*t* = 2.65, *df* = 120, *p* = 0.009). Notably, 61% of our patient population also experience anxiety and participants with anxiety were more likely to also have vulvodynia than those without (78% vs. 55%, *p* = 0.002). No other factors differed significantly among participants with or without vulvodynia. Logistic regression analysis (Table 2) indicates individuals with anxiety are 2.8 times more likely to have vulvodynia than those without. However, women with a higher BMI were less likely to have vulvodynia.

**Table 2.** Logistic Regression Analysis

Variable	Estimate	***p***	OR (95% CI)
Yeast	−0.229	0.515	0.7 (0.4–1.58)
BMI	−0.065	0.014	0.9 (0.88–0.98)
Anxiety	1.032	0.002	2.8 (1.42–5.51)

CI, confidence interval; OR, odds ratio.

## Discussion

The findings in this study showed no association between vulvodynia and yeast infection. These results deviate from some prior studies that have found a positive association between self-reported history of yeast infection and vulvar pain. One potential explanation for this deviation may be that patients in prior studies are often reporting history of presumed yeast infection. Relying on reported history of prior yeast infections without laboratory confirmation may result in over-reporting in a population that has increased vulvar sensitivity at baseline and may over-report symptoms when compared with a baseline population.

The frequency with which patients who self-report a history of yeast infections but may have been treated without laboratory confirmation of yeast in these studies is not known. Furthermore, empiric treatment may lead patients to overperceive the frequency of their infections. For example, Reed et al.^12^ described that 36.2% of patients in a cohort who screened positive for vulvodynia by questionnaire had been previously diagnosed with yeast infection, yet there was no difference in symptoms improvement among those who received treatment and those who did not.

The authors noted that this suggests that patients should be followed to confirm resolution of symptoms. Expanding on this, the symptom persistence could potentially be explained by empiric treatment of presumed yeast that was not present, co-occurrence of yeast and vulvodynia, or recurrent yeast infections. However, without culture confirmation of infection, an association is somewhat speculative.

Limitations of our study include absence of data regarding self-reported history of yeast infections. As this information was not consistently available, we did not include it in our analysis; however, this would be an area of interest for future study to determine how many patients would also report a history of yeast infection in this population. We also acknowledge that a negative yeast result at the time of presentation to our clinic does not necessarily exclude prior confirmed yeast infections and that this study cannot account for any prior confirmed yeast infections in this patient cohort.

Other limitations include cross-sectional descriptive methodology without ability to establish causal relationships that limit understanding of trends over time^13^ and self-reporting inherent in survey methodology that impacts generalization of findings.^14^ It is also worth noting that these data were collected from patients in a multidisciplinary clinic that cares for patients seeking routine gynecological care, as well as specialized care for chronic pelvic pain, pelvic floor and vulvar disorders. Clinical pathways took into account common subtypes of vulvodynia such as provoked and unprovoked primary and secondary vulvodynia, but severity was not evaluated as part of our practice.

Yeast infection was reported in a study by Donders et al.^15^ to be more often identified by microscopy in women with mild forms of vulvodynia, however, this could not be assessed as part of this study due to the fact that it is not part of routine practice to rate the severity of vulvodynia. Objective assessment of patient improvement in contrast is routinely evaluated as part of follow-up visit intake, although it was not specifically evaluated as part of this study. Within a 6-month period during which this study took place, there was notable improvement between the patient's first follow-up visit (Mean = 3.25, standard deviation [SD] = 1.12) and last visit (Mean = 2.82, SD = 1.13), *t*(207) = 5.47, *p* ≤ 0.0001, among all patients presenting to the clinic using the Patient Global Impression of Improvement.^16^

All patients are screened with the GAD-7 and PHQ-9 at their initial visit, which allowed us to collect these data. For a portion of the patients in this study, survey responses were not captured in a structured data field due to a change in PRO vendor. However, among 148 patients in our cohort where the data could be extracted to evaluate, GAD-7 and PHQ-9 questionnaires were completed in 91% and 76% of patients, respectively.

Questionnaires were sent to patients in advance of their appointment, and those who were unable to complete surveys in advance were asked to complete them on tablets while awaiting their appointment. The PHQ-9 was only available to be completed on the day of the appointment for patient safety, which may account for the difference in response rate between GAD-7 and PHQ-9 in our cohort. For the purposes of this study, we considered a patient positive for anxiety based on reported history and/or elevated GAD score. There did not appear to be the same association with depression as for anxiety in the vulvodynia population.

Other associations with vulvodynia identified included low BMI and anxiety. Although BMI data would be available in most clinics, the routine use of screening tools for mood disorders is not standard in all women's health clinics. Patient referral base included private practice clinics as well as a federally qualified health center. Thus, the patient population in this study included a broad range of socioeconomic backgrounds. In terms of racial and ethnic differences, patients who identified as white were the predominant group represented in this study followed by Hispanic and/or Latina, with minimal representation of black, indigenous, and women of color.

Although this is reflective of our local population as a whole, the findings may not be widely applicable, such as in populations with a high prevalence of African American patients. Further studies are needed to determine whether there are differences by racial and/or ethnic background. In addition, prospective trials are needed to investigate the causal effects of candida on vulvar pain.

## Conclusion

The findings in this study showed no association between vulvodynia and yeast infection, a divergence from prior studies. In addition, vulvodynia was associated with low BMI and anxiety. Further studies are needed to better differentiate whether these results would differ between different subtypes of vulvodynia and correlate with patient-reported history of yeast infection. To improve generalizability, further study is needed among women of color with vulvodynia. Finally, further research is needed to better understand association and causality between VVC infections and vulvodynia in women.

## Author Note

The article is submitted in accordance with current author guidelines for the sole consideration of *Journal of Women's Health* and the material has not been published in any form previously.

## Abbreviations Used

BMI: body mass index
CI: confidence interval
EHR: electronic health record
GAD: Generalized Anxiety Disorder
ICD: International Classification of Disease
IQR: interquartile range
IRB: institutional review board
ISSVD: International Society for the Study of Vulvovaginal Disease
LPV: localized provoked vulvodynia
OR: odds ratio
PCR: polymerase chain reaction
PHQ: patient health questionnaire
PRO: patient-reported outcome
SD: standard deviation
STIs: sexually transmitted infections
VVC: vulvovaginal candida
